# Reliability of the MacArthur scale of subjective social status - Brazilian Longitudinal Study of Adult Health (ELSA-Brasil)

**DOI:** 10.1186/1471-2458-12-1096

**Published:** 2012-12-20

**Authors:** Luana Giatti, Lidyane do Valle Camelo, Jôsi Fernandes de Castro Rodrigues, Sandhi Maria Barreto

**Affiliations:** 1 Post-graduate Program in Public Health, School of Medicine, Universidade Federal de Minas Gerais, Belo Horizonte, MG, Brazil; 2 Research Group on Chronic and Occupational Diseases – GERMINAL, Universidade Federal de Minas Gerais, Belo Horizonte, MG, Brazil; 3 School of Nutrition, Universidade Federal de Ouro Preto, Morro do Cruzeiro s/n, Ouro Preto, MG, 35400-000, Brazil

**Keywords:** Subjective social status, Reproducibility, Cohort study, ELSA-Brasil

## Abstract

**Background:**

The MacArthur Scale of Subjective Social Status intend to measure the subjective social status using a numbered stepladder image. This study investigated the reliability of the MacArthur scale in a subsample of the Brazilian Longitudinal Study of Adult Health (ELSA-Brasil).

**Method:**

Three scales were employed using different references: 1) the overall socioeconomic position; 2) the socioeconomic situation of the participant’s closer community; 3) the workplace as a whole. A total of 245 of the ELSA participants from six states were involved. They were interviewed twice by the same person within an interval of seven to fourteen days. The reliability of the scale was assessed with weighted Kappa statistics and intraclass correlation coefficient (ICC), with their respective 95% confidence interval (CI).

**Results:**

Kappa values were 0.62(0.58 to 0.64) for the society ladder; 0.58(0.56 to 0.61) for the community-related ladder; and 0.67(0.66 to 0.72) for the work-related ladder. The ICC ranged from 0.75 for the work ladder to 0.64 for the community ladder. These values differed slightly according to the participants’ age, sex and education category.

**Conclusion:**

The three ladders showed good stability in the test-retest, except the community ladder that showed moderate stability. Because the social structure in Brazil is rapidly changing, future qualitative and longitudinal studies are needed to confirm and understand the construct underlying the MacArthur Scale in the country.

## Background

The relationship between socioeconomic conditions and the population’s health is a well established fact. Objective socioeconomic position indicators such as income, occupation and education have been used to measure individuals’socioeconomic position [1,2]. Despite the strength and importance of objective indicators of socioeconomic position, they do not explain the entire process by which socioeconomic disadvantages contribute to increased morbidity and mortality.

The subjective aspects of social position appear to be relevant in order to understand the causal mechanism between social inequalities and health problems [3,4]. Subjective social status reflects the relative perception that individuals have of their place in the social hierarchy. This indicator express the feelings of individuals belonging to a certain social stratum, and capture current and past socioeconomic situation, future prospects, family resources, life opportunities, the way people experience society and how they perceive themselves in relation to others [4-6].

Despite the undeniable importance of measures of subjective social status, there is still considerable debate concerning their subjective nature. It is not entirely clear what this indicator really captures. Most researchers inquire about the influence of psychological factors on how individuals identify themselves in the social hierarchy ladder. Another debated aspect concerns the reference group for comparison. In general, associations between social status and health indicators tend to be stronger when the reference group is more general, i.e., the society as a whole [7-9]. Nonetheless, it is also important to consider the temporal comparison that individuals establish between their present situation with their past one [10,11].

Subjective social status can be measured with different instruments. The more conventional method consists in applying questionnaires that investigate how the person perceives his or her place in the social hierarchy, with categorical response options like working class, middle class or upper class [12-15]. This kind of instrument requires perceptions of the class system, otherwise instruments use visual representations. The MacArthur Scale of Subjective Social Status is a pictorial representation that uses a symbolic ladder, developed to capture the common sense of social status based on usual socioeconomic status indicators. It has the additional advantage to allow comparisons between studies conducted in different populations [16].It has been used in large epidemiological European [4,7,17] and US studies [16] and found to be significantly associated with health status, independently of objective socioeconomic indicators.

The Brazilian Longitudinal Study of Adult Health (ELSA-Brasil) has included a wide range of measures of socioeconomic position at baseline, among which is the MacArthur Scale of Subjective Social Status. The ELSA-Brasil study is an occupational cohort. For this reason, a third scale was included to assess how individuals see themselves on a social ladder which uses as reference groups people who occupy the highest and the lowest positions in their working place. The assessment of job related social status in Brazil could add a new and important dimension to the study on sources of social inequalities in health among Brazilian population. No previous study has evaluated the MacArthur Scale of Subjective Social Status in Brazil, thus making the present study imperative.

ELSA-Brasil quality control activities included reliability studies in order to assess the stability of measurements in subsamples and also to verify whether the stability differ according to individuals characteristics. Verifying the reliability of the MacArthur Scale of Subjective Social Status in the population of ELSA-Brasil may provide elements that can show the adequacy of using this instrument to assess the subjective social status The aim of this study was to evaluate the test-retest reliability of the MacArthur scale and its adaptation for the measurement of subjective social status at work among civil servants participants in the ELSA-Brasil. Furthermore, it investigated whether the test-retest reliability of the scales differ according to participants characteristics (age, sex and schooling).

## Methods

### Study population

The ELSA-Brasil study is a prospective multicenter study developed in institutions of higher education and research, located in six Brazilian states: Minas Gerais, São Paulo, Rio de Janeiro, Espírito Santo, Bahia and Rio Grande do Sul. The main objectives of this study are to investigate the incidence and progression of diabetes and cardiovascular diseases and to examine the biological, behavioral, environmental, occupational, psychological and social factors associated with these diseases and their complications, seeking to build a causal model that contemplates their inter-relations. Civil servants, age 35 to 74 – including current and retired employees of each of the seven public institutions of higher education and research participating in the study – were considered eligible to be subjects for the research. Data collection occurred between August 2008 and December 2010. Details of the study design have been described elsewhere [18].

For the study of test-retest reliability, a convenience sample of 245 participants from the six ELSA Research Centers (RCs) were selected according to pre-established quotas for sex (males: 50%; female: 50%), age (35–44: 13%; 45–54: 36%; 55–64: 38%; 65–74: 13%) and occupation (unskilled: 35%; technical/clerical: 30%; faculty and professional staff: 35%). In this study, age was grouped into two categories: 35–54 and 55–74 years. There was no statistically significant difference between the ELSA-Brasil study population and the study sample composition.

The first application of the scale occurred during the face-to-face interview, which is part of the ELSA-Brasil baseline data-gathering. At the end of this phase, participants were invited to answer the scales again. The second application of the instrument was administered by the same interviewer who conducted the first one, within an interval of seven to fourteen days. Both first and second interviews were carried out at the subjects’ workplace or at the ELSA Research Center during the baseline examination. The present study was completed between November 2009 and September 2010.

### Instrument

**Figure 1:**
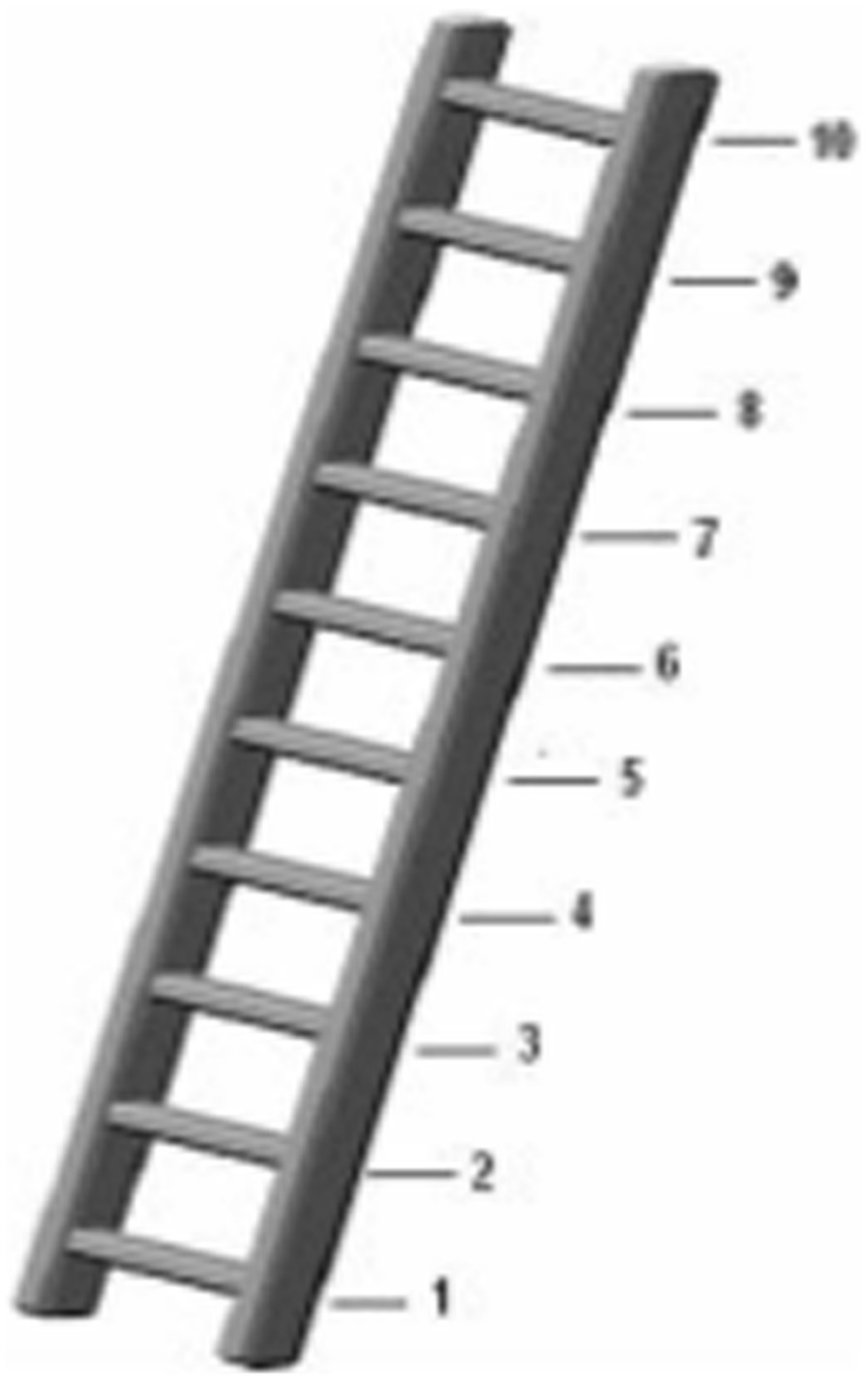
Illustration of the ladder used in the “MacArthur” Scale of Subjective Social Status applied to participants of ELSA-Brasil at baseline, 2008–2010.

The MacArthur Scale of Subjective Social Status is presented in a ladder format with 10 steps (Figure 1).There are two versions of the ladder that have distinct references to which individuals can compare themselves. The society ladder is a global measure of subjective social status and is related to the individual’s place in the social hierarchy.

References used in this scale are objective indicators such as income, education and occupation. The community ladder, on the other hand, assesses how individuals see themselves in the ladder considering the community where they live in; those who present higher living standards are at the top of the ladder and those who present lower ones are at the bottom [19].

In the ELSA-Brasil study individuals were asked to choose the step where they are located in the social hierarchy (society ladder) or in their community (community ladder). Because the ELSA-Brasil study is an occupational cohort, we also created a new ladder to assess how participants perceive themselves in the occupational hierarchy. This third ladder replicates the same instructions used for the other ones, replacing the top and bottom references with high and low valued jobs (work ladder).

The scale was translated by two researchers fluent in English and underwent testing and adjustments. In order to detect major flaws, the scale, as well as the full study questionnaire, were subjected to three consecutive pre-testing at the six RCs, totaling 73 pre-test interviews with ineligible workers and staff people: 16 in São Paulo’s RC, 10 in Minas Gerais’ RC, 12 in Rio de Janeiro’s RC, 11 in Bahia’s RC, 11 in Rio Grande do Sul’s RC and 12 in Espírito Santo’s RC. After revising the instructions, the scales were again tested in three pilot studies, totaling 360 interviews with individuals who bear very similar personal characteristics to ELSA-Brasil population in the six centers. At the end of this process, final adjustments were made to the ladders, especially to the community ladder. Because the word “community” in Brazil tends to be associated with “slums”, it was necessary to change it to “neighborhood”.

Study participants were shown the scale by means of a card, and invited to answer the following questions:

"Consider that the ladder that I am showing you represents the place that people occupy in society. At the top of this ladder are the people who have more money, more education and better jobs. At the bottom of the ladder are the people who have less money, less education and worse jobs (jobs with less recognition) or are unemployed."

"The higher you consider yourself in this ladder, the closer you will be to the people who are at the top of the ladder, and the lower, closer you will be to people who find themselves at the bottom. Where would you place yourself on this ladder?"

For the community ladder the introduction was replaced by the following explanations:

"Following the same logic of the previous question, now consider this ladder as representing where people stand in their neighborhoods. People define neighborhood in different ways; please define it in whatever way is most meaningful to you. At the top of the ladder are the people who have the highest standing in their neighborhood. At the bottom are the people who have the lowest standing in their neighborhood. Considering the living standard of people in your neighborhood, where would you place yourself on this ladder?"

The instructions of the work ladder were:

"Finally, following the same logic, consider this ladder as representing where people stand in their workplace. People define work in different ways; please define it in whatever way is most meaningful to you. On the top of the ladder are people who have the most valued jobs, as the director or the president, for example. At the bottom of the ladder are people who hold the less valued jobs. Considering your work, where would you place yourself on this ladder?"

For the reliability study we used the following data: sex, age group (35–54, 55–74 years) and education (up to high school graduation and college graduate or more).

### Data analysis

Data input into the Epi-Info program were made by independent double data entry. Data analysis was performed using Stata version 10.0. First we described the characteristics of the study population using frequency distributions and measures of central tendency. Then a descriptive analysis of the scales of subjective social status was generated by means of the average and standard deviation, median, 25^th^ and 75^th^ percentiles and the range of variation in first and second application. Next, we assessed the reliability of the scales by means of intraclass correlation coefficent (ICC) and the weighted Kappa statistic. For all statistics, 95% confidence intervals were estimated. We also calculated the ICC and Kappa statistics for each of the scales stratified by sex, age and education. Weights defined by STATA were used to calculate the weighted Kappa statistics.

Altman’s criteria was used for the interpretation of concordance calculated by the Kappa statistic: a) poor: -1 to 0.20; b) reasonable: 0.20 to 0.40; c) moderate: 0.41 to 0.60; d) good: 0.61 to 0.80; and e) very good: 0.81 to 1.00 [20]. ICC was evaluated according to Landis & Koch criteria: a) poor < 0, b); weak: 0–0,20, c) probable: 0,21-0,40, d) moderate: 0,41-0,60, e) substantial: 0,61-0,80, d) almost perfect: 0,81-1,00 [21].

### Ethical issues

ELSA-Brasil research protocol was approved by the Research Ethics Committee of UFMG, FIOCRUZ, UFES, UFBA and UFRGS and also by the National Research Ethics Committee. An informed consent was signed by all participants.

## Results

Of the 245 study participants, 51% were women, 59% were between the age of 35 and 54 years and 53% had completed a university education (Table 1).

**Table 1:** Distribution of the test-retest reliability study population according to sex, age and education and subjective social status

	**Number**	**Percentage**
**Sex**		
Male	120	49.0
Female	125	51.0
**Age range (years)**		
35-54	144	58.8
55-74	101	41.2
**Education**		
≤ High school	115	46.9
College or University	130	53.1
**Subjective scale of social status - Society**		
1(Low status)	1	0.4
2	4	1.63
3	8	3.3
4	19	7.8
5	36	14.7
6	52	21.2
7	64	26.1
8	38	15.5
9	15	6.1
10(High status)	8	3.3
**Subjective scale of social status - Community**		
1(Low status)	2	0.8
2	5	2.0
3	6	2.5
4	14	5.7
5	33	13.5
6	45	18.4
7	51	20.8
8	50	20.4
9	32	13.1
10(High status)	7	2.9
**Subjective scale of social status - Work**		
1(Low status)	3	1.2
2	4	1.6
3	5	2.0
4	11	4.5
5	37	15.1
6	30	12.2
7	54	22.0
8	69	28.2
9	26	10.6
10(High status)	6	2.5

ELSA-Brasil, 2008–2010.

The mean of the three ladders ranged from 6.4 to 6.8 in the test and from 6.8 to 6.9 in the retest. The median of the three ladders in both the test and retest was 7.0. The 25^th^ and 75^th^ percentiles were 6.0 and 8.0 respectively, except for the society ladder which had a median of 7.0 and 25^th^ and 75^th^ percentiles of 5.0 and 7.0 in the first interview. According to the criteria used to evaluate the values of the Kappa statistic, the society and work ladders had good reproducibility (>0.60), while the community ladder showed moderate reproducibility (=0.58).For all scales, the ICC indicated substantial test-retest reliability that ranged from 0.64 for the community ladder to 0.75 for the work ladder (Table 2).

**Table 2:** Mean, standard deviation, median, 25th and 75th percentiles, range, intraclass correlation coefficient (ICC) and 95% confidence intervals, weighted Kappa statistics index and the 95% confidence interval of the subjective social status scale

		**Society**	**Community**	**Work**
Mean (std. dev.)	Test	6.40 (1.71)	6.68 (1.82)	6.77 (1.79)
	Retest	6.75 (1.54)	6.84 (1.65)	6.89 (1.64)
Median and (25 & 75 percentiles)	Test	7 (5 and 7)	7 (6 and 8)	7 (6 and 8)
	Retest	7 (6 and 8)	7 (6 and 8)	7 (6 and 8)
Range	Test	1 – 8	1 – 10	1 – 10
	Retest	2 – 10	1 – 10	1 – 10
ICC (95% CI)		0.67 (0.39-0.96)	0.64 (0.34-0.93)	0.75 (0.50-1.00)
Weighted Kappa statistic (95% CI)		0.62 (0.58-0.64)	0.58 (0.56-0.61)	0.67 (0.66-0.72)

ELSA-Brasil, 2008–2010.

95% CI: 95% confidence interval.

The Kappa and ICC statistics for each ladder are presented in Table 3 and are broken up according to sex, age and education. The Kappa statistic ranged from 0.54, for the community ladder among younger subjects, to 0.71, for the work scale among the older ones. The ICC varied from 0.56 (for the society ladder among the less educated and the community ladder among the youngest) to 0.82 (work ladder for the oldest).The stratified analysis showed differences statistically significant by sex, age and education, as the Kappa statistic confidence intervals did not overlap. Reproducibility of the ladders was better among older individuals (all scales), those with higher education (society) and men (work).

**Table 3:** Intraclass correlation coefficient (ICC) with 95% confidence intervals and weighted Kappa statistic with 95% confidence interval of the subjective social status scale according to sex, age and educational level

	**Society**		**Community**		**Work**	
	**ICC (95% CI)**	**Kappa (95% CI)**	**ICC (95% CI)**	**Kappa (95% CI)**	**ICC (95% CI)**	**Kappa (95% CI)**
**Sex**						
Male	0.65 (0.35-0.95)	0.59 (0.54-0.65)	0.69 (0.43-0.95)	0.61 (0.58-0.70)	0.77 (0.54-1.00)	0.70 (0.70-0.79)
Female	0.71 (0.43-0.99)	0.64 (0.60-0.68)	0.57 (0.22-0.92)	0.55 (0.46-0.66)	0.72 (0.44-1.00)	0.63 (0.54-0.67)
**Age (years)**						
35-54	0.62 (0.30-0.95)	0.54 (0.48-0.57)	0.56 (0.22-0.90)	0.54 (0.44-0.59)	0.70 (0.40-1.00)	0.63 (0.57-0.65)
55-74	0.74 (0.50-0.99)	0.70 (0.65-0.74)	0.73 (0.48-0.98)	0.64 (0.62-0.68)	0.82 (0.63-1.01)	0.71(0.68-0.78)
**Education**						
≤ High School	0.56 (0.22-0.89)	0.54 (0.48-0.55)	0.64 (0.34-0.95)	0.57 (0.53-0.60)	0.71 (0.44-0.98)	0.64 (0.59-0.65)
College/University	0.72 (0.43-1.00)	0.65 (0.57-0.72)	0.58 (0.25-0.91)	0.56 (0.50-0.61)	0.76 (0.49-1.03)	0.66 (0.62-0.68)

ELSA-Brasil, 2008–2010.

95% CI: 95% confidence interval.

## Discussion

The present study evaluated the test-retest reliability of the MacArthur Scale of Subjective Social Status and its adaptation to measure the subjective social status based on the occupational hierarchy in Brazilian civil servants. Test-retest reliability range from moderate for community ladder to good for society and work ladders according to Kappa statistics; and according to the ICC the reliability was substantial for the three ladders. Low reliability may influence the performance of a measure, contribute to participant misclassification and attenuate associations with other variables [22]. In our results the lower reliability was observed for the community ladder which showed moderate kappa statistics.

Difference in reliability according to individual characteristics may result in differential levels of misclassification. The reliability of MacArthur scale in this study ranged in line with age, education and sex. We found that test-retest reliability of the three scales tended to be better among older individuals. It is possible that individuals aged 55 or more are more stable in different dimensions of their social position and hence more consistent in their answers about their position in the three ladders. Likewise, this might occur among higher educated participants. Goodman et al. [23] found variation in the ICC values according to sex and age, but unfortunately, these authors did not show the confidence interval to better evaluate the statistical significance.

The work scale exhibited the best performance in test-retest reliability, especially among men and older participants. This might be explained by the fact that they all belong to the same career (civil servants at university or research public institutions) which has both, top and bottom positions, clearly defined. Thus, compared to the society and community ladders, it has much less cultural, social and economic variability. This scale presented low reliability among women. It is possible that gender differences in ELSA-Brasil are smaller than in the society as whole, but it still exists, as women and men continue to live and work within a gendered society [24]. It is possible that the women's perception of their position at work might be more unstable as their job situations have less social recognition and social position is more likely to be influenced by their spouses or family’s position.

Using a different method, from that which was used in the present study, Operario et al. [25] examined the 6-month reproducibility of the society ladder in a subsample of 191 US adults and found a Spearman’s correlation coefficient of 0.62 (p < 0.01). Because ICC is more rigorous than Sperman’s correlation coefficient to measure reliability [26], our result (ICC = 0.67) could be considered more trustworthy than that of the US study. However, the US study was carried out in a younger (18 years and more), more socially diverse and exposed to greater social mobility than our study population. Otherwise, Goodman et al. [23] found higher ICCs as compared to ours (0.73 for the society ladder and of 0.79 for the community ladder), but they evaluated the reproducibility among adolescents using an adapted version of the MacArthur scales of Subjective Social Status that assess familial placement in US society and personal placement in the school community becoming the comparison with our results less appropriate.

Our results show that the use of the MacArthur scales and its adaptation for the measurement of subjective social status at work in the ELSA-Brasil is appropriate as they showed good reliability. This is important because this alternative indicator has been reported as being a cognitive averaging of standard markers of socioeconomic measures [4] and can capture changes in socioeconomic positions over the life course better than objective indicators of social position [27]. This alternative indicator seems to work similarly to the self-rated health as a measure of overall health [28,29], as it captures a balance between measured and unmeasured factors that influence one’s position in the social context. Consequently, the use of the subjective social status may help to identify a relevant dimension of the social position that cannot be measured only by income, education or occupation [30]. For this reason, we believe that the use of this indicator in the Brazilian context can bring new elements to understand the complexity of the expression of social inequalities that translate into health inequalities in our country.

The goal of empirical research is to obtain measures that are as accurate and reproducible as possible [31]. A strength of this paper is the use of a test-retest interval which was not too short nor too long (one to two weeks). This period has been considered appropriate because it minimizes the chances of any substantial change in individual’s life and is long enough for them to forget what they replied in the first interview [32].

The translation process was carried out initially by two of the researchers, SMB & LG. Because the instructions are quite simple and straightforward we did not carry on a back translation. For this reason, we are sure that the final version is as close as possible to the original one. It is important to highlight that in our study, for the community ladder, we use the word “neighborhood” instead of “community”. This was done because this is the closest word to community in the Brazilian cultural context, and is not confused with slum. However, this may limit the comparison of our findings with other studies with regard to the reliability of the community ladder.

The ELSA-Brasil consists of a very specific population of civil servants and has no intention of representing the Brazilian population. But, the sociodemographic characteristics of the sample participating in this work is similar to those of the entire cohort, lending support to the reliability of these scales and its future use to investigate associations between subjective social status and health, both cross-sectionally and longitudinally.

## Conclusions

The three ladders evaluated showed good stability between the test and the retest, with the exception of the community ladder which showed moderate stability. To further understand this alternative indicator and the meaning of a given position in the social hierarchy at the ELSA-Brasil study, the association of qualitative studies with ongoing longitudinal studies and the dialogue with other disciplines, such as sociology and economics, is important.

## Competing interests

The authors declare that they have no competing interests.

## Authors’ contributions

LG and SMB made substantial contributions to the study’s conception, acquisition of data, analysis and interpretation of data, have been involved in drafting the manuscript and have given final approval of the version to be published. LVC and JFCR were involved in reviewing the literature and helped draft the manuscript. All authors read and approved the final manuscript.

## Pre-publication history

The pre-publication history for this paper can be accessed here:

http://www.biomedcentral.com/1471-2458/12/1096/prepub
